# Rating dental arch relationships and palatal morphology with the EUROCRAN index on three different formats of dental casts in children with unilateral cleft lip and palate

**DOI:** 10.1007/s00784-015-1595-0

**Published:** 2015-10-13

**Authors:** Alijda J. Sabelis, Mette A. R. Kuijpers, Rania M. Nada, Yu-Ting Chiu, Ewald M. Bronkhorst, Anne Marie Kuijpers-Jagtman, Piotr S. Fudalej

**Affiliations:** Department of Orthodontics and Craniofacial Biology, Radboud University Medical Center, Philips van Leydenlaan 25, NL 6525 EX Nijmegen, The Netherlands; Department of Developmental and Preventive Sciences, Faculty of Dentistry, Kuwait University, Kuwait City, State of Kuwait; Department of Dentistry and Craniofacial Center, Chang Gung Memorial Hospital, Taipei, Taiwan; Department of Cariology and Preventive Dentistry, Radboud University Medical Center, Nijmegen, The Netherlands; Department of Orthodontics and Dentofacial Orthopedics, University of Bern, Bern, Switzerland; Department of Orthodontics, Palacky University Olomouc, Olomouc, Czech Republic

**Keywords:** Cleft palate, Treatment outcome, Dental models, EUROCRAN index, Dental arch relationship

## Abstract

**Background:**

The EUROCRAN index has been used in inter-center studies to assess dental arch relationship (DAR) and palatal morphology (PM) in children with unilateral cleft lip and palate (UCLP). For this type of inter-center research, a scoring method that could be performed over the internet would be the most effective. Therefore, the aim of this study was to investigate the reliability of application of the EUROCRAN index on 3D digital models or photographs of plaster models instead of using plaster models.

**Methods:**

The EUROCRAN reference models were presented in three formats: plaster models, 2D photographs of plaster models, and 3D digital models. Plaster models of children with UCLP (*n* = 45) were rated. Of each case, all three formats were rated by six calibrated observers in random order. The strength of agreement of the ratings was assessed with kappa statistics. Concordance among observers was evaluated with the intra-class correlation coefficient (ICC).

**Results:**

The ICC showed a good inter-observer agreement for the DAR and poor inter-observer agreement for the PM. Intra-observer agreement for the DAR was moderate to very good, yet for the PM poor to moderate. Comparison between the three formats per observer for the DAR was good or very good and for the PM moderate to poor.

**Conclusions:**

The overall results show that the EUROCRAN index is an acceptable and reliable scoring method for the DAR on plaster models, 2D photographs of plaster models, and 3D digital models. However, due to the small range of deviations in palatal morphology between the cases in our study, the PM component of the index was difficult to assess.

**Clinical relevance:**

In clinical audits and inter-center studies, plaster models can be substituted by 2D photographs of plaster casts or 3D digital models when grading treatment outcome with the EUROCRAN index.

## Introduction

In patients with unilateral cleft lip and palate (UCLP), dental arch relationship is assessed with several different methods such as the GOSLON yardstick [1], the Huddart/Bodenham crossbite scoring method and its modification [2–4], incisal overjet measuring method [5], and the 5-year-olds’ index score [6]. However, scoring with some of these grading systems [2–5] may fail to take into account the severity of the malocclusion as a whole and has the potential for underestimating the discrepancy between the arches [7]. Among the rating systems mentioned above, the GOSLON yardstick [1] and the 5-year-olds’ index [6] are the more comprehensive rating scales, which categorize dental arch relationship in anterior-posterior, vertical, and transverse dimensions in older and younger patients, respectively. Both indices have five categories, from grade 1 equivalent with excellent treatment outcome to grade 5 meaning very poor outcome and need for orthognathic surgery.

The EUROCRAN index was developed as a spin-off of the EUROCRAN project by Katsaros et al. [unpublished data], as it was felt that the GOSLON yardstick—developed nearly 30 years earlier—and the 5-year-olds’ index—published in 1997—were not very well suitable for grading more recent cohorts of CLP patients in which the treatment outcomes for cleft lip and palate patients are generally better than in the past [8]. The EUROCRAN index is a modification of the GOSLON yardstick and 5-year-olds’ index. Furthermore, it is extended with a score for palatal morphology [9–11]. It is assumed that scar tissue that develops over the denuded palatal bone after palatoplasty contributes to growth disturbances [12]. Therefore, evaluation of palatal morphology should be part of the evaluation of treatment outcome. This indicates that an index, which also includes rating of palatal morphology, may be of importance. The EUROCRAN index is the only index, which assesses two components: the occlusal relationship in all three planes of space (including displacement of the lesser segment on the cleft side) and the palatal morphology (see Table 1) [8–11].

**Table 1:** Grade allocation according to the EUROCRAN index [Katsaros et al. unpublished data, 9, 14]

Grades	Dental arch relationship
1	(a) Apical base relationship skeletal Class I or Class II. Both central incisors positive overjet and overbite. Note: If both incisors have a positive overjet and overbite but the incisor relationship was achieved by obvious dental compensation/orthodontic treatment, the case is grade 2.
	(b) Apical base relationship skeletal Class I or Class II. No overbite but overjet markedly increased. Note: If there is no overbite and the overjet is not markedly increased, the case is grade 2.
2	Apical base relationship skeletal Class I. Non-cleft incisor in positive overjet and overbite. Tilting or derotation would achieve stable positive overjet and overbite of the incisor on the cleft side. Note: The case is grade 3 if there is a moderate open bite.
3	(a) Apical base relationship edge to edge or mild Class III. One or both central incisors edge to edge or in anterior crossbite. Tilting or derotation would not achieve a stable positive overjet and overbite (i.e., the proclined tooth would relapse), may include moderate open bite. Note: If both incisors have an edge to edge relationship but the skeletal Class is III (i.e., incisor relationship was achieved by dental compensation/orthodontic treatment), the case is grade 4
4	(a) Apical base relationship Class III Both centrals in anterior crossbite or one in anterior crossbite with the other edge to edge Central incisors may or may not be in contact with the lower incisors. (b) As grade 3 but with a marked open bite
Grades	Palatal morphology
1	Good anterior and posterior height; minor surface irregularities (bumps, crevices); Nil or minor deviation of arch form
2	Moderate anterior and posterior height; moderate surface irregularities (bumps, crevices); Moderate deviation of arch form (e.g., segmental displacement)
3	Severe reduction in palate height; severe surface irregularities (bumps, crevices); severe deviation in arch form, e.g., “hourglass” constriction)
	The worst feature of the three suggests the initial score. This may be modified up or down depending on how good the other features are. If good arch form was achieved by means of orthodontic treatment, the case is graded lower.

Until now, the EUROCRAN index has been applied to plaster models only [13–15]. Currently, 3D digital dental models are common practice. They have great advantages over plaster models in archiving, viewing, and retrieval and can be accessed at any time and at any distance for diagnostic, clinical, and research purposes [16–22]. Yet, 3D digital models are associated with disadvantages. They cannot be held and viewed in the same way as plaster models, and familiarization with their use takes time. Furthermore, although a digital model is 3D, the image viewed on screen is only 2D [23, 24]. Many studies have been performed using the GOSLON yardstick and the 5-years-olds’ index on plaster models, whereas studies utilizing photographs of plaster models and 3D digital models are quite rare [22, 25–28]. Dogan et al. [22] found that the GOSLON scoring on photographs of dental casts and 3D digital models showed a high reliability when compared with ratings on plaster models of the dental arch relationship of UCLP patients. Chawla et al. [7] investigated the reliability of four different formats of the 5-years-olds’ index: plaster models, colored acrylic models, black and white photographs, and 3D digital models. They found that the 3D digital models and digital photographs are reliable alternatives to plaster models for the 5-year-olds’ index. This has not been tested for the EUROCRAN index.

Therefore, the aim of this study is to investigate the reliability of using 3D models or photographs of plaster models instead of plaster models for rating dental arch relationship and palatal morphology in children with UCLP with the EUROCRAN index. The hypothesis to be tested is that there is no difference between the gradings of the three different formats.

## Materials and methods

The use of anonymous data gathered during routine patient care is in accordance with Dutch law on medical research. A written statement of the institutional review board (IRB) was obtained stating that this study does not fall within the remit of the Medical Research Involving Human Subjects Act (WMO). Therefore, the present investigation could have been carried out without an individual approval by an accredited research ethics committee. No formal waiver of approval by the IRB was obtained. All parents/legal guardians gave written informed consent for the use of images of their children in the study.

### Material

Plaster models (Plas-M) of 45 patients with non-syndromic complete UCLP with a mean age of 9 years (SD 1.6) were used in this study. Some patients were treated orthodontically in the past with a simple removable appliance in the upper jaw; no fixed appliances were used. From the plaster models, 2D digital photographs (2D-M) and 3D digital models (3D-M) were obtained.

**Fig. 1:**
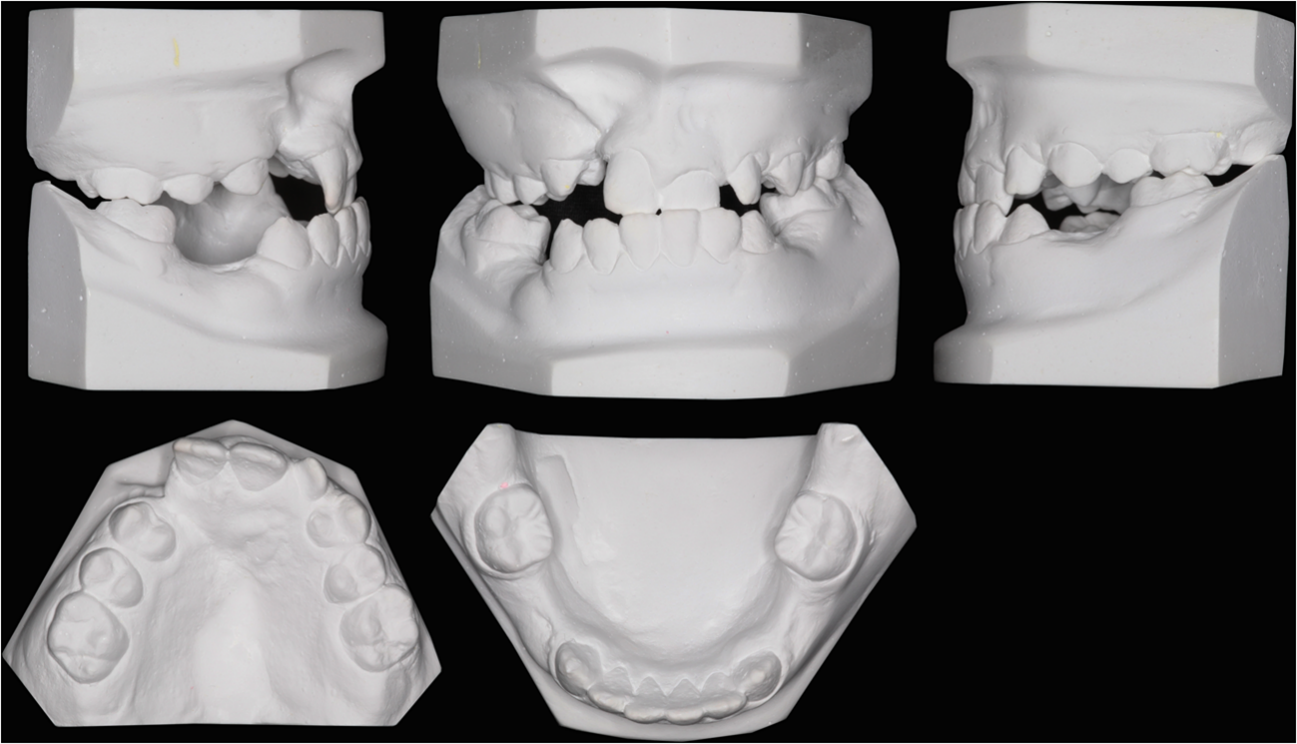
One set of photographs of the plaster models of a patient with UCLP

The 2D-Ms were made with a Canon EOS 5D (Canon Inc, Tokyo, Japan) camera and a 100 mm lens. The lens-object distance was 30 cm. A set of five views of the plaster models was made with a black background (Fig. 1). Subsequently, the images were loaded into PowerPoint2007 (Microsoft Corp., Redmond, WA, USA). Two types of slides were prepared for rating: One slide contained five views of the plaster models and the other slide contained only an enlarged palatal view of the maxillary plaster models. The slides were displayed and rated on a laptop.

**Fig. 2:**
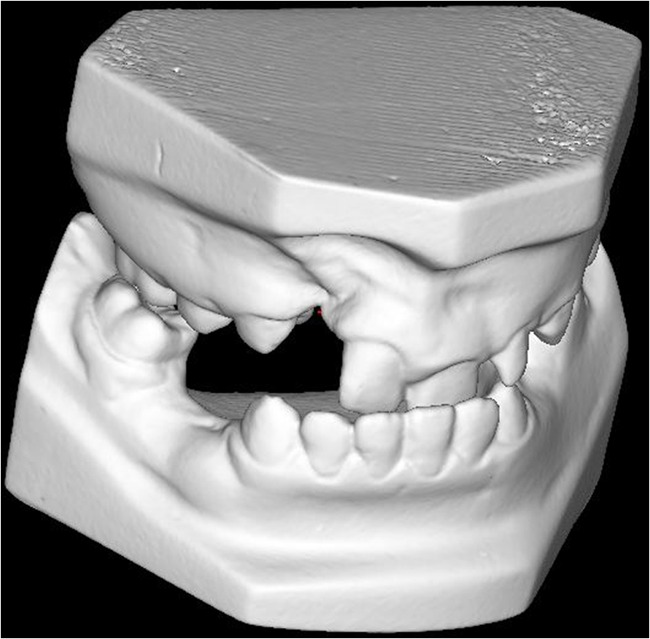
Digital model for the patient shown in Fig. 1. By viewing the digital model from different angles, the transverse occlusion can be clearly assessed, showing a crossbite in this patient

In order to obtain 3D-Ms, all Plas-M were digitized (Orthoproof, Doorn, The Netherlands) according to a standardized procedure. The 3D-Ms were displayed on a laptop using the program Digimodel® (Ortholab BV, Doorn, The Netherlands) (Fig. 2). The observers were instructed to manipulate the 3D-Ms with the software enhancement tools (i.e., allowing for zooming and rotation) according to their own preference.

### Method

The EUROCRAN index [9, 13, 14] was used to rate treatment outcome on three kinds of media formats: Plas-Ms, 2D-Ms, and 3D-Ms. According to the index, two components are rated separately: (1) *dental arch relationship* (DAR; grades from 1 to 4, in which 1 means a very good treatment outcome and 4 corresponds to a poor outcome and necessity for orthognatic surgery) and (2) *palatal morphology* (PM; from 1, meaning good morphology, to 3, meaning poor morphology) (Table 1). Six calibrated observers were involved—four orthodontists experienced in treatment of cleft palate patients (O1, O2, O3, and O4), one final year postgraduate orthodontic trainee (O5), and one second year postgraduate orthodontic trainee (O6). An extensive calibration session was performed separately for the DAR and PM. During calibration, sets of three different formats of 20 patients not belonging to the experimental group were used.

The 45 sets of Plas-Ms, 45 sets of 2D-Ms, and 45 sets of 3D-Ms were coded with a random number. To prevent a possible influence of fatigue on the results, the rating material was divided into three groups comprising 15 Plas-Ms, 15 2D-Ms, and 15 3D-Ms each. These groups differed in type and in order of formats of individual patients. For example, Plas-Ms of a given patient were included in group 1, photographs of plaster models in group 2, and digital models in group 3. The order of rating in the groups was as follows: in group 1, Plas-M → 2D-M → 3D-M; in group 2, 2D-M → 3D-M → Plas-M; and in group 3, 3D-M → Plas-M → 2D-M. Thus, each component of the EUROCRAN index was assessed on 135 sets of formats. Observers took a 30-min break between rating the groups 1 and 2 and between rating the groups 2 and 3. Moreover, the rating was arranged in such a way that the material scored just before and just after the break came from different patients.

The DAR was graded in the morning session. After a 1-h break, the PM was rated. Anchor (i.e., reference) models that demonstrate examples for all different grades and all types of formats were available for comparison. Also, each observer had a copy of the EUROCRAN index rating guidelines according to Katsaros et al. [unpublished data] and described in earlier studies [9, 13, 14].

To evaluate the intra-observer agreement, 21 randomly selected data sets were reassessed after 2 weeks.

## Statistical analysis

The EUROCRAN index utilizes a categorical scale, which implies the use of the kappa statistics for analysis of observer performance. Because the EUROCRAN index is also an ordinal scale, its scores can be treated as nominal scores [29]. In that case, the reliability coefficient (RCoef), the duplicate measurement error (DME), and the systematic difference between paired observations are assessed for method error analysis. We used both approaches. For each format and between the formats, intra-observer performance was analyzed by calculating kappa statistics, RCoef, DME, and the difference between paired observations. Concordance among observers during rating of each of the three formats was evaluated with the intra-class correlation coefficient (ICC). Strength of agreement assessed with kappa statistics was interpreted according to Landis and Koch [30]: poor (kappa < 0.2), fair (0.21–0.4), moderate (0.41–0.6), good (0.61–0.8), and very good (0.81–1).

## Results

### Inter-observer performance for the three formats

Table 2 show that, irrespective of the format, the ICCs for the DAR were considerably higher than for the PM. This suggests a good inter-observer reliability for grading the DAR and relatively poor inter-observer reliability for scoring the PM. Within each component of the EUROCRAN index, the ICCs for each format were comparable. No differences were found in inter-observer performance for plaster models and the two other formats (2D-M and 3D-M) (*p* > 0.4).

**Table 2:** Inter-observer performance for plaster models (Plas-M), 2D digital photographs of plaster models (2D-M), and 3D digital models (3D-M) expressed as intra-class correlation coefficient (ICC) with 95 % confidence intervals

		Plas-M	2D-M	3D-M
DAR	ICC	0.849	0.846	0.866
	[95 % CI]	[0.784…0.904]	[0.779…0.901]	[0.806…0.915]
PM	ICC	0.258	0.258	0.260
	[95% CI]	[0.145…0.405]	[0.145…0.405]	[0.147…0.407]

*DAR* dental arch relationship, *PM* palatal morphology, *Plas-M* plaster models, *2D-M* 2D digital photographs of plaster models, *3D-M* 3D digital models, *ICC* intra-class correlation coefficient, *CI* confidence interval

### Intra-observer performance for DAR and PM

Intra-observer agreement for the Plas-M, 2D-M, and 3D-M is presented in Tables 3 and 4. The reliability coefficients ranged from 0.822 to 0.975 for DAR (Table 3) and from −0.069 to 0.833 for PM (Table 4).

**Table 3:** Intra-observer performance (six observers, O1 to O6) for the dental arch relationship (DAR) component of the EUROCRAN index

	Observer	Reliability	Mean diff.	95 % CI of mean diff	*p* value	DME	Kappa
Plas-M	O1	0.923	0.143	[−0.075…0.360]	0.186	0.338	0.667
	O2	0.822	0.000	[−0.322…0.322]	1.000	0.500	0.745
	O3	0.931	0.095	[−0.103…0.294]	0.329	0.309	0.745
	O4	0.857	0.190	[−0.083…0.464]	0.162	0.425	0.489
	O5	0.867	0.000	[−0.288…0.288]	1.000	0.447	0.679
	O6	0.880	0.238	[−0.046…0.523]	0.096	0.442	0.599
2D-M	O1	0.922	0.000	[−0.204…0.204]	1.000	0.316	0.733
	O2	0.965	0.095	[−0.042…0.232]	0.162	0.213	0.871
	O3	0.877	0.000	[−0.249…0.249]	1.000	0.387	0.612
	O4	0.907	0.000	[−0.204…0.204]	1.000	0.316	0.726
	O5	0.925	−0.143	[−0.360…0.075]	0.186	0.338	0.673
	O6	0.945	0.190	[0.007…0.374]	0.042	0.285	0.732
3D-M	O1	0.876	−0.095	[−0.380…0.189]	0.493	0.442	0.662
	O2	0.958	0.000	[−0.144…0.144]	1.000	0.224	0.872
	O3	0.923	0.095	[−0.103…0.294]	0.329	0.309	0.742
	O4	0.863	0.190	[−0.042…0.423]	0.104	0.362	0.802
	O5	0.975	0.095	[−0.042…0.232]	0.162	0.213	0.865
	O6	0.946	0.190	[0.007…0.374]	0.042	0.285	0.735

*Plas-M* plaster models, *2D-M* 2D digital photographs of plaster models, *3D-M* 3D digital models, *diff* difference, *CI* confidence interval, *DME* duplicate measurement error

**Table 4:** Intra-observer performance (six observers, O1 to O6) for the palatal morphology (PM) component of the EUROCRAN index

	Observer	Reliability	Mean diff.	95 % CI of mean diff	*p* Value	DME	Kappa
Plas-M	O1	0.014	0.048	[−0.221…0.316]	0.715	0.417	0.013
	O2	0.315	0.048	[−0.221…0.316]	0.715	0.417	0.246
	O3	0.333	0.000	[−0.288…0.288]	1.000	0.447	0.257
	O4	0.672	0.095	[−0.103…0.294]	0.329	0.309	0.600
	O5	0.833	0.048	[−0.127…0.223]	0.576	0.272	0.755
	O6	0.258	0.143	[−0.155…0.441]	0.329	0.463	0.074
2D-M	O1	0.200	−0.143	[−0.360…0.075]	0.186	0.338	0.173
	O2	0.447	0.381	[0.154…0.607]	0.002	0.352	0.333
	O3	−0.069	0.286	[−0.041…0.612]	0.083	0.507	−0.059
	O4	0.414	0.286	[0.075…0.496]	0.010	0.327	0.292
	O5	0.408	0.095	[−0.150…0.341]	0.428	0.381	0.400
	O6	0.636	−0.048	[−0.223…0.127]	0.576	0.272	0.632
3D-M	O1	0.315	0.048	[−0.221…0.316]	0.715	0.417	0.125
	O2	0.395	0.429	[0.198…0.659]	0.001	0.359	0.270
	O3	0.408	0.238	[−0.007…0.483]	0.056	0.381	0.364
	O4	0.400	0.190	[0.007…0.374]	0.042	0.285	0.276
	O5	0.485	−0.048	[−0.274…0.179]	0.666	0.352	0.483
	O6	0.614	0.190	[−0.083…0.464]	0.162	0.425	0.317

*Plas-M* plaster models, *2D-M* 2D digital photographs of plaster models, *3D-M* 3D digital models, *diff* difference, *CI* confidence interval, *DME* duplicate measurement error

Regardless of the format, intra-observer agreement for the DAR was good or very good (0.8 ≥ kappa > 0.6 or kappa > 0.8, respectively) for all except two observations (observers 4 and 6 for grading Plas-M). For the PM, intra-observer agreement was good (kappa > 0.6) only for 2 out of 18 observations (observer 5 for Plas-M and observer 6 for 2D-M). In the remaining situations, intra-observer agreement was poor to moderate.

### Comparison between the three formats per observer for DAR and PM

The intra-observer agreement between the three formats is shown in Tables 5 and 6. The reliability coefficients per observer ranged from 0.783 to 0.968 for DAR (Table 5) and from −0.085 to 0.640 for PM (Table 6).

**Table 5:** Comparison of intra-observer performance between the three formats (plaster models (Plas-M), 2D digital photographs of plaster models (2D-M), and 3D digital models (3D-M)) per observer for the dental arch relationship (DAR) component of the EUROCRAN index

	Observer	Reliability	Mean diff.	95 % CI of mean diff	*p* Value	DME	Kappa
Plas-M vs. 2D-M	O1	0.852	0.048	[−0.221…0.316]	0.715	0.417	0.460
	O2	0.914	−0.143	[−0.360…0.075]	0.186	0.338	0.734
	O3	0.931	−0.095	[−0.294…0.103]	0.329	0.309	0.704
	O4	0.809	−0.095	[−0.380…0.189]	0.493	0.442	0.590
	O5	0.867	0.000	[−0.288…0.288]	1.000	0.447	0.672
	O6	0.903	−0.048	[−0.274…0.179]	0.666	0.352	0.517
Plas-M vs. 3D-M	O1	0.822	0.190	[−0.119…0.500]	0.214	0.481	0.524
	O2	0.927	−0.095	[−0.294…0.103]	0.329	0.309	0.823
	O3	0.968	−0.095	[−0.232…0.042]	0.162	0.213	0.734
	O4	0.783	−0.048	[−0.352…0.257]	0.748	0.473	0.516
	O5	0.870	−0.048	[−0.352…0.257]	0.748	0.473	0.548
	O6	0.947	0.048	[−0.127…0.223]	0.576	0.272	0.637

*Plas-M* plaster models, *2D-M* 2D digital photographs of plaster models, *3D-M* 3D digital models, *diff* difference, *CI* confidence interval, *DME* duplicate measurement error

**Table 6:** Comparison of intra-observer performance between the three formats (plaster models (Plas-M), 2D digital photographs of plaster models (2D-M), and 3D digital models (3D-M)) per observer for the palatal morphology (PM) component of the EUROCRAN index

	Observer	Reliability	Mean diff.	95 % CI of mean diff	*p* Value	DME	Kappa
Plas-M vs. 2D-M	O1	0.200	0.143	[−0.075…0.360]	0.186	0.338	0.262
	O2	0.640	0.095	[−0.103…0.294]	0.329	0.309	0.564
	O3	0.333	0.000	[−0.288…0.288]	1.000	0.447	0.176
	O4	0.201	−0.095	[−0.341…0.150]	0.428	0.381	0.282
	O5	0.599	0.238	[−0.007…0.483]	0.056	0.381	0.375
	O6	0.356	0.190	[−0.083…0.464]	0.162	0.425	0.287
Plas-M vs. 3D-M	O1	0.389	−0.048	[−0.274…0.179]	0.666	0.352	0.487
	O2	0.640	0.095	[−0.103…0.294]	0.329	0.309	0.418
	O3	0.096	0.048	[−0.289…0.384]	0.771	0.523	0.112
	O4	−0.085	−0.143	[−0.404…0.118]	0.267	0.405	0.349
	O5	0.367	0.333	[0.034…0.633]	0.031	0.465	0.098
	O6	0.388	−0.190	[−0.532…0.151]	0.258	0.530	0.296

*Plas-M* plaster model, *2D-M* 2D digital photographs of plaster models, *3D-M* 3D digital models, *diff* difference, *CI* confidence interval, *DME* duplicate measurement error

Intra-observer agreement for comparison of Plas-M with 2D-M and 3D-M in grading the DAR demonstrated that concordance was good to very good in 6 out of 12 comparisons (kappa > 0.6) and moderate in six comparisons (kappa ≤ 0.6). The level of concordance was considerably lower for grading PM—in three comparisons, intra-observer agreement was moderate, in six—fair, and in the remaining—poor.

## Discussion

For inter-center studies or studies that require rating by external observers, the observers or plaster models or both must travel to do the rating. This inevitably involves extra costs, inconvenience, and risk of damage to the plaster models. A more convenient approach would be to substitute the plaster models by photographs of plaster models or 3D digital models and perform the scoring over the internet. Such a grading session would be cost-effective [25] and relatively easy to arrange. To test its feasibility, we investigated the reliability of using 3D-M or 2D-M of plaster models instead of Plas-M when assessing treatment outcome with the EUROCRAN index in children with UCLP.

The EUROCRAN index is a fairly new tool for assessing treatment result in patients with UCLP. The index grades give an indication of the overall treatment outcome for a certain center. It may also supplement information obtained of the same individuals for craniofacial growth using 2D or 3D cephalometry [28]. The overall results for the intra- and inter-observer reliability show that the EUROCRAN index is acceptable and reliable for scoring the DAR. These findings are in concordance with earlier studies [13, 14] that reported values of kappa for intra-observer agreement ranging from 0.49 to 0.91 (moderate to very good agreement), i.e., comparable with the values from the current investigation. Our results partially disagree with findings of Patel [15]. She found poorer intra-observer agreement during assessment of the DAR component of the EUROCRAN index, yet she assessed patients at the age of 5 years, whereas we examined 9-year-olds.

The present results demonstrate that it is possible to replace plaster models with 2D-Ms or 3D-Ms for grading the DAR component of the EUROCRAN index. It is in keeping with other studies, which assessed reliability of grading occlusion in patients with cleft lip and palate based on formats alternative to plaster models [7, 22–28].

The reliability of assessment of the palatal morphology component is questionable for all formats. This finding is in agreement with the results of the study by Patel [15] and partly in concordance with earlier findings from our group [13, 14]. Both research groups found a lower reliability for scoring PM than DAR, but the reliability of the scoring PM obtained by Fudalej et al. [13, 14] was considerably higher than in the present study. A reason of this discordance may be the fact that there were only small differences in palatal vault morphology in the 45 cases rated in the present study, whereas the range of palatal dysmorphology may have been larger in the groups assessed earlier [13, 14]. Because of the low agreement in grading the PM, we suggest to modify the PM grading scale and/or guidelines. Additionally, adding a second photograph of the palate for grading of palatal height in the PM assessment on 2D photographs could improve the effectiveness of the grading.

## Conclusion

The overall results show that the EUROCRAN index is an acceptable and reliable scoring method for the dental arch relationships on plaster models, 2D photographs of plaster models, and 3D digital models. However, due to the small range of deviations in palatal morphology between the cases in our study, the PM component of the index was difficult to assess.
